# Which explanations do clinicians prefer? A comparative evaluation of XAI understandability and actionability in predicting the need for hospitalization

**DOI:** 10.1186/s12911-025-03045-0

**Published:** 2025-07-16

**Authors:** Laura Bergomi, Giovanna Nicora, Marta Anna Orlowska, Chiara Podrecca, Riccardo Bellazzi, Caterina Fregosi, Francesco Salinaro, Marco Bonzano, Giuseppe Crescenzi, Francesco Speciale, Santi Di Pietro, Valentina Zuccaro, Erika Asperges, Paolo Sacchi, Pietro Valsecchi, Elisabetta Pagani, Michele Catalano, Chandra Bortolotto, Lorenzo Preda, Enea Parimbelli

**Affiliations:** 1 Department of Electrical, Computer and Biomedical Engineering, Via Ferrata 5, Pavia, 27100 Italy; 2 Department of Computer Science, Systems and Communication, University of Milano-Bicocca, Milan, Italy; 3 Emergency Medicine Unit and Emergency Medicine Postgraduate Training Program, Department of Internal Medicine, IRCCS Policlinico San Matteo Foundation, University of Pavia, Pavia, Italy; 4 PhD Program in Experimental Medicine, University of Pavia, Pavia, Italy; 5 Department of Clinical, Surgical, Diagnostic and Pediatric Sciences, University of Pavia, Pavia, Italy; 6 Infectious Diseases Unit, Fondazione IRCCS Policlinico di Pavia, Pavia, Italy; 7 Radiology Department, Fondazione IRCCS Policlinico San Matteo, Pavia, Italy

**Keywords:** User study, Questionnaire, Human-AI collaboration, Interpretability, Think-aloud protocol, Clinical decision-making

## Abstract

**Background:**

This study aims to address the gap in understanding clinicians’ attitudes toward explainable AI (XAI) methods applied to machine learning models using tabular data, commonly found in clinical settings. It specifically explores clinicians’ perceptions of different XAI methods from the ALFABETO project, which predicts COVID-19 patient hospitalization based on clinical, laboratory, and chest X-ray at time of presentation to the Emergency Department. The focus is on two cognitive dimensions: *understandability* and *actionability* of the explanations provided by explainable-by-design and post-hoc methods.

**Methods:**

A questionnaire-based experiment was conducted with 10 clinicians from the IRCCS Policlinico San Matteo Foundation in Pavia, Italy. Each clinician evaluated 10 real-world cases, rating predictions and explanations from three XAI tools: Bayesian networks, SHapley Additive exPlanations (SHAP), and AraucanaXAI. Two cognitive statements for each method were rated on a Likert scale, as well as the agreement with the prediction. Two clinicians answered the survey during think-aloud interviews.

**Results:**

Clinicians demonstrated generally positive attitudes toward AI, but high compliance rates (86% on average) indicate a risk of *automation bias*. *Understandability* and *actionability* are positively correlated, with SHAP being the preferred method due to its simplicity. However, the perception of methods varies according to specialty and expertise.

**Conclusions:**

The findings suggest that SHAP and AraucanaXAI are promising candidates for improving the use of XAI in clinical decision support systems (DSSs), highlighting the importance of clinicians’ expertise, specialty, and setting on the selection and development of supportive XAI advice. Finally, the study provides valuable insights into the design of future XAI DSSs.

**Supplementary Information:**

The online version contains supplementary material available at 10.1186/s12911-025-03045-0.

## Background

For decades, Artificial Intelligence (AI) has captivated researchers and practitioners in the medical field. Today, AI technologies are reaching a level of maturity that allows them to be integrated into clinical practice, particularly in radiology, the area where most AI-powered medical devices have received Food and Drugs Administration (FDA) approval [1, 2]. As AI tools transition from research to clinical application, it is essential that clinicians, the end-users, understand the reasoning behind an AI algorithm’s results [3, 4]. This knowledge would enable them to incorporate AI predictions into clinical decision-making in a well-informed manner.

Explainable AI (XAI) is a subfield of AI focusing on the development of methods able to explain the AI reasoning process in a human-understandable way [5]. Indeed, XAI has been recently outlined as a requirement for AI applications in the medical domain by the European Union AI Act, the first binding regulation of AI [6]. Under this regulation, any AI system to be used in clinical practice will be required to provide users with explanations regarding their reasoning process. However, while the integration of XAI holds promise, it also raises important concerns. Notably, seemingly persuasive explanations (even for incorrect advice) can foster unwarranted trust in AI systems. This phenomenon, referred to as the “white box paradox”, may result in null or even harmful effects [7, 8].

Researchers have already developed various XAI methods, each coming with a specific way of representing the “explanation” [9]. For example, SHapley Additive exPlanations (SHAP) [10], probably the most widely used XAI method, conveys explanations in terms of feature importance. AraucanaXAI [11], on the other hand, locally generates tree-based explanations for specific predictions, that can be subsequently translated into IF-THEN rules. While these methods are *post-hoc*, and able to explain the prediction of a “black box” AI system like deep neural networks, some AI approaches, like Bayesian Networks or Logistic regression, are explainable-by-design and do not need additional “XAI layer” to be interpretable. Indeed, researchers have also advocated that in high stakes applications, such as medicine, only interpretable models should be employed [12].

As there is no one-size-fits-all method to convey explainability, studies investigating end-user perceptions and preferences toward different forms of explanations are required. Available studies are usually focusing on the opinions and acceptance of clinicians toward AI [13–15]. A recent survey of 23 clinicians reported that XAI, in the form of SHAP explanations, usually increases confidence in predictions of a publicly available AI system [16, 17]. However, there is a lack of studies investigating how different forms of explanations are perceived by various categories of healthcare professionals, including clinicians [18]. Moreover, currently, studies focus either on signals [19] or images [20, 21]. User attitudes toward XAI on tabular data, which remains the predominant format for presenting clinical information, are still under-investigated. In this work, we aim to investigate clinicians’ perceptions of different XAI methods through a user study. These methods are used to interpret the output of machine learning (ML) models, trained within the ALFABETO (ALl FAster BEtter TOgheter) project to predict COVID-19 patients’ hospitalization, and applied to patient tabular data including information on the patient’s clinical characteristics, laboratory tests and chest X-rays. Given three different XAI tools, we conduct a questionnaire-based experiment to collect clinicians’ cognitive evaluation of explanations and their preferences. In particular, we focus on *understandability* and *actionability* as two relevant dimensions to evaluate the goodness of explanations, adapted from [22]. *Understandability* refers to how easily a user can intuitively grasp the explanation. *Actionability* refers to the extent to which an explanation enables a user to make an informed decision or take appropriate action in response to the case at hand. By goodness of explanations, we refer to the overall quality of an explanation generated by an XAI tool, as perceived by clinicians, in terms of how effectively it supports these two key dimensions. An explanation is considered good if it is both easy to comprehend intuitively (*understandability*) and effectively supports informed clinical decision-making or appropriate action (*actionability*).

Based on the data collected, we aim to answer the following research questions (RQ):

1. Is there any correlation between *actionability* and *understandability* of explanations?
2. Do clinician characteristics (e.g., expertise or specialty) affect how explanations are perceived and preferred?

The main finding concerns the influence of expertise, specialty, and the setting in which the clinician makes decisions, on the selection and development of supportive XAI advice.

## Methods

### Machine learning classifiers and XAI methods

Within the ALFABETO project [23, 24], ML classifiers have been previously developed to predict whether a COVID-19 patient in electronic health records requires hospitalization or home care treatment, predicted respectively to be in the ‘Hospital’ or ‘Home’ class. All the patients in the ALFABETO project were treated in the emergency room of the IRCCS Policlinico San Matteo Foundation in Pavia, Italy. The project focuses on clinical and laboratory data collected at the time each patient presents to the emergency department, along with features extracted from chest X-ray images taken upon their arrival.

Clinical data are age, gender, and presence of respiratory issues (cough, breathing difficulties, chronic obstructive pulmonary disease, and respiratory failure). The laboratory data are White Blood Cell count (WBC) and C-reactive Protein (CRP). In addition, the following comorbidities from the past medical history of the patients are used: hypertension, type 2 diabetes mellitus, cardiovascular disease, chronic renal failure, stroke, ischemic heart disease, atrial fibrillation, heart failure, dementia, and active cancer in the last 5 years. The numerical radiomic features extracted from chest X-ray images are Consolidation, Infiltration, Edema, Effusion, and Lung Opacity. In this study, we use previously developed classifiers that predict whether a patient should be hospitalized based on all the aforementioned features as inputs. Details regarding the ML classifiers and their performance can be found in previous publications [24]. Specifically, the classifiers include an explainable-by-design Bayesian Network and a “black-box” ensemble model, Gradient Boosting.

A key challenge of “black-box” models lies in their complexity, which often makes it difficult even for domain experts to interpret how the results are derived. To enhance the interpretability of such AI systems for human decision-makers, various strategies can be employed [25]. In this context, we select SHAP [10] and AraucanaXAI [11] as XAI *post-hoc* local models to provide users with insights (i.e. explanations) into the predicted class of the “black box” model for each patient. SHAP assigns an importance score borrowed from coalitional game theory (Shapley values) to input features to show how they influence a model’s prediction for a specific patient [10]. Positive contributions push the model toward the ‘Hospital’ class, while negative contributions toward the ‘Home’ class. The features are displayed in order of importance, and the total sum of these contributions determines the final probability of a ‘Hospital’ prediction. On the other hand, AraucanaXAI generates a local set of neighboring instances by augmenting the original training data through oversampling. It then grows an explainable-by-design decision tree, that can be translated into IF-THEN rules. The explanation is derived by navigating the tree based on the feature values of the patient’s case being analyzed [11]. We evaluate explanation fidelity by measuring the level of agreement between the two XAI *post-hoc* models and the outputs of the “black-box” model. SHAP explanations showed perfect fidelity with 100% agreement (50 out of 50 cases used in this study), while AraucanaXAI explanations showed 96% agreement (48 out of 50 cases).

**Fig. 1:**
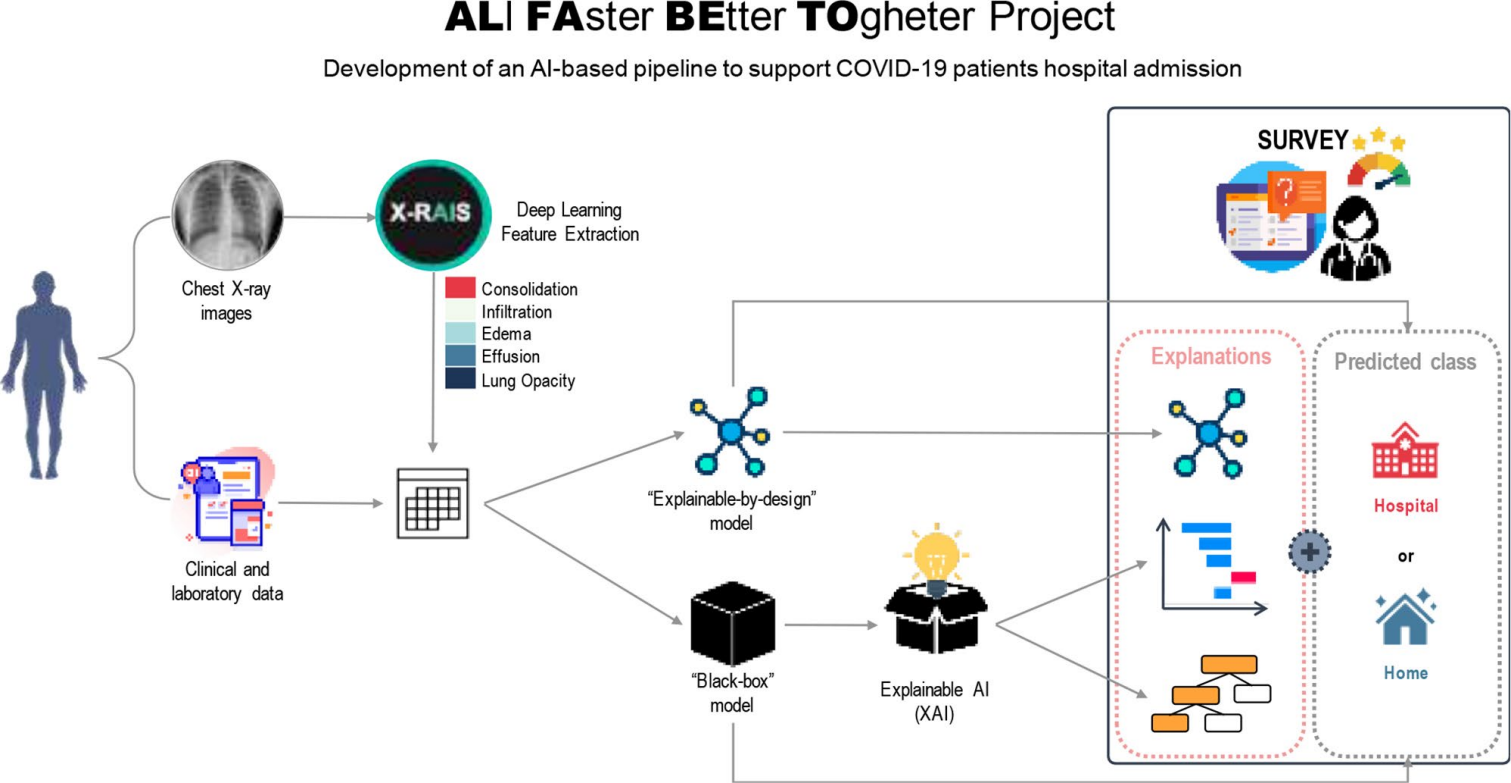
Overall logical architecture of this study. In the ALFABETO project, two models are employed to predict whether COVID-19 patients require hospitalization or home care: an “explainable-by-design” model and a “black-box” model, both using clinical, laboratory, and chest X-ray features. The primary contribution of this study lies in the incorporation of an Explainable AI (XAI) component, which enhances the interpretability of the “black-box” model’s predictions. Therefore, the survey assesses how the intended end-users, i.e. clinicians, evaluate the explanation –in terms of its understandability and actionability in supporting their decision-making process– and whether they agree with the prediction

The overall logical architecture of the study is illustrated in Fig. 1.

### XAI survey design

**Fig. 2:**
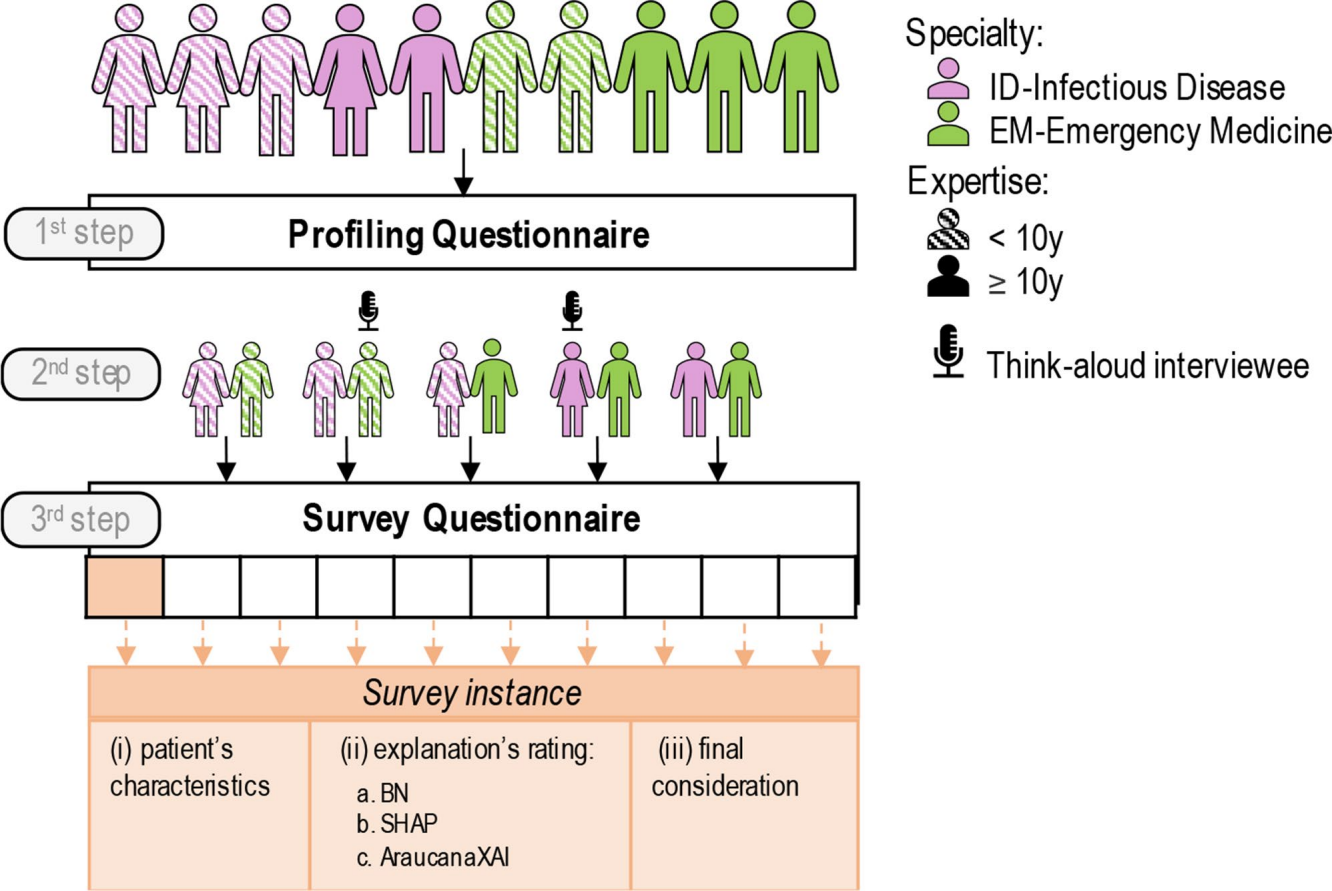
Workflow of the study design. The study involves 10 clinicians–5 specialists in infectious diseases (ID) and 5 specialists in emergency medicine (EM). (1st step) Participants profile’s characteristics are collected through the Profiling Questionnaire, especially regarding attitudes towards AI, specialty and years of experience. This latter information is used to (2nd step) match clinician pairs, who (3rd step) receive a unique version of the Survey Questionnaire, composed by 10 survey instances. Two clinicians complete the survey during a Think-aloud interview

To capture clinicians’ perceptions and preferences regarding XAI, we designed and implemented the study using the KoboToolbox online platform. The study consists of two questionnaires (the pdf version is shown in the Supplementary Materials): a preliminary participant profiling questionnaire–*Profiling Questionnaire*, followed by the main *Survey Questionnaire*. The study aligns with Levels 4 and 5 in the evidence hierarchy for AI and XAI empirical studies [26], as it involves “retrospective real-world cases considered by real practitioners in simulated/laboratory settings”. The workflow of the study design is shown in Fig. 2.

The study involves 10 experienced clinicians from the IRCCS Policlinico San Matteo Foundation in Pavia, Italy (the same hospital from which the ALFABETO project’s patients were recruited)—5 specialists in infectious diseases (ID) and 5 specialists in emergency medicine (EM), who voluntarily participate in the study. The two specialties (ID and EM) are selected because of their prevalent involvement in the management of the pandemia. Before administering the questionnaires, an educational session is conducted to ensure that all clinicians have a consistent understanding of the project and are better equipped to interpret the explanations presented during the survey. Such training is performed with a recorded video explaining (i) the classification task solved by the classifiers, including the input features, and (ii) the different XAI approaches and how to interpret them. Following this session, we gathered their profiles through the *Profiling Questionnaire*, collecting information on their years of experience as a medical specialist, specialty (EM or ID), gender, and attitudes towards AI. To assess this last aspect, we included five statements (reported in Table 1) adapted from the Perceived Usefulness scale of the Technology Acceptance Model (TAM) [27], a framework commonly employed in research to explore how users accept and use new technologies. On the basis of years of experience and specialty, we matched clinicians—one from EM and one from ID, resulting in 5 clinician pairs.

Finally, we delivered the *Survey Questionnaire* in which each clinician is presented with 10 patient cases from the ALFABETO test set (50 distinct patient cases in total), without knowing the true class, i.e. the decision made during the pandemic (whether the patient was in fact hospitalized or not). Even without showing the true class, the clinicians are aware that some of the cases are correctly predicted, meaning they are accurately classified as ‘Hospital’ (True Positive–TP) or ‘Home’ (True Negative–TN), while the remaining are incorrectly predicted, being misclassified as ‘Hospital’ (False Positive–FP) or ‘Home’ (False Negative–FN). More precisely, we selected cases for the questionnaires in such a way that the TPs and TNs are shown before the FPs and FNs to prevent negative priming against the explanations and predictions [28, 29]. We showed each clinician exactly 5 cases of correct prediction and 5 cases of incorrect prediction.

The following information is available for each patient’s case (i.e. *survey instance*): (i) the patient’s characteristics in tabular format (classifiers input features), (ii. a) the prediction and the explanation from the explainable-by-design Bayesian Network (BN), (ii. b) the prediction of the ‘black-box’ model (Gradient boosting) and its consistent explanations as SHAP and (ii. c) AraucanaXAI tree. Clinicians are asked to rate two cognitive statements for each explanation (i.e. BN, SHAP, and AraucanaXAI, presented in random order) on a Likert scale from 1-strongly disagree to 6-strongly agree, specifically:

S1. I find the explanation intuitively understandable.

S2. The explanation helps me take a proper decision about the case at hand.

These two statements are adapted from the Explanation Satisfaction Scale (ESS) [22], and represent *understandability* (S1) and *actionability* (S2). Along with these two dimensions, ESS includes several cognitive dimensions to help developers and researchers assess the goodness of and satisfaction with explanations. We decided to focus our analysis on these two main dimensions, which we consider the most fit to our analysis, to avoid overwhelming clinicians with too many questions for each case. In addition to that, (iii) there are two final questions at the end of each *survey instance* about the agreement with the predicted class and the most suitable explanation in the classification task:

FQ1. Overall, do you agree with the predicted class for the patient?

FQ2. Overall, which explanation method did you find most suitable and intuitive in the classification task for the patient?

Each of the 50 *survey instances* is evaluated independently by two clinicians (i.e. clinician pair). Each clinician evaluates 10 *survey instances*, resulting in 100 *survey instances* overall.

Finally, research questions are answered using statistical hypothesis testing: RQ1 with the Spearman correlation coefficient ρ (and associated p-values) and RQ2 with the Wilcoxon signed-rank test and Mann-Whitney U test for paired and unpaired comparisons, respectively. Significance is assessed at the 95% confidence level.

### Think-aloud protocol

The think-aloud protocol, widely recognized in software engineering and cognitive psychology, is highly applicable to decision-making processes. It has been previously employed to evaluate decision-making in healthcare settings [30–33]. During a think-aloud session, the user is expected to verbalize thoughts, feelings, and actions that arise while performing a task. This unstructured approach helps explore and identify issues and insights that the investigator may not have considered, providing feedback and decision subtleties that can only be captured in real-time as decisions are being made [34].

Two clinicians–one from EM and one from ID– completed the survey questionnaire while following the think-aloud protocol in their usual clinical setting, assisted by one cognitive psychology trained researcher of the team (M.A.O.). To facilitate smooth verbalization and avoid discrepancies due to varying English proficiency, they speak in their native language, i.e. Italian [34]. The whole session is recorded with their written consent. At the start, the clinicians are encouraged to verbalize thoughts, feelings, and impressions as they complete the questionnaire. The protocol specifies no interaction with them during the survey unless they remain silent for about 10 s. After data collection, the recordings are automatically transcribed using the advanced transcription tool TurboScribe (https://turboscribe.ai/), which also provides timestamps. The transcripts are then carefully proofread to ensure accuracy and consistency with the original audio. The inclusion of timestamps enables precise segmentation and time measurement, allowing the calculation of time spent on each patient case and explanation after manual annotation. In this study, time is the most effective indirect measure of usability, as well as the best proxy for cognitive load –a key factor in usability studies and evaluation of XAI explanations [35]. The final goal of the think-aloud sessions is to elicit the factors that influence clinicians’ perceptions and thus make the results of the evaluation more robust.

## Results

### Clinicians’ attitudes towards AI

Table 1 shows the profiling data collected through the *Profiling Questionnaire*, stratified by specialty.

**Table 1:**
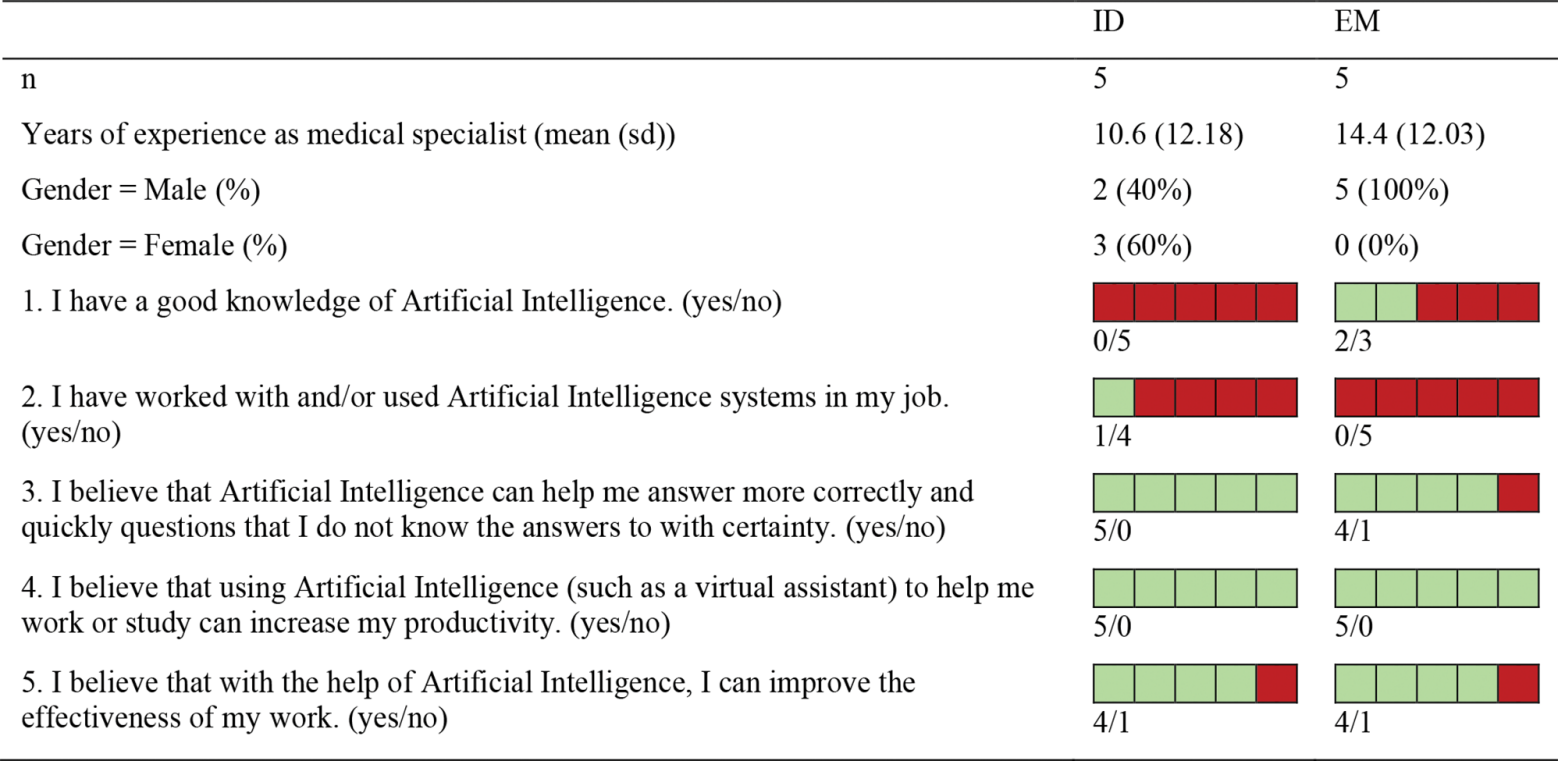
Clinicians’ profiling data stratified by specialty – ID vs. EM. Clinicians’ attitudes towards AI are indicated by statements numbered 1 to 5: ‘yes’ answers are highlighted in green, while ‘no’ answers in red

The analysis of clinicians’ attitudes towards AI, based on their responses to the *Profiling Questionnaire* (i.e. statements numbered 1 to 5 in Table 1), reveals two key dimensions: familiarity with AI and the positive or skeptical stance towards it. Familiarity with AI is determined by a ‘yes’ response to either statement 1 or 2. Of the clinicians surveyed, three demonstrate some familiarity with AI by responding affirmatively to at least one of these. Perceived skepticism towards AI is determined by a ‘no’ response to statement 3 or 5. Two out of ten clinicians express skepticism, with one clinician answering ‘no’ to both statements. On the other hand, a ‘yes’ response to these statements indicates a positive attitude towards AI. The remaining five clinicians are classified as unfamiliar with AI but have a generally positive attitude toward its use in healthcare. All the clinicians answer positively to statement 4, indicating a consensus on the beneficial role of AI in supporting their work or studies and enhancing productivity.

### XAI survey results

**Fig. 3:**
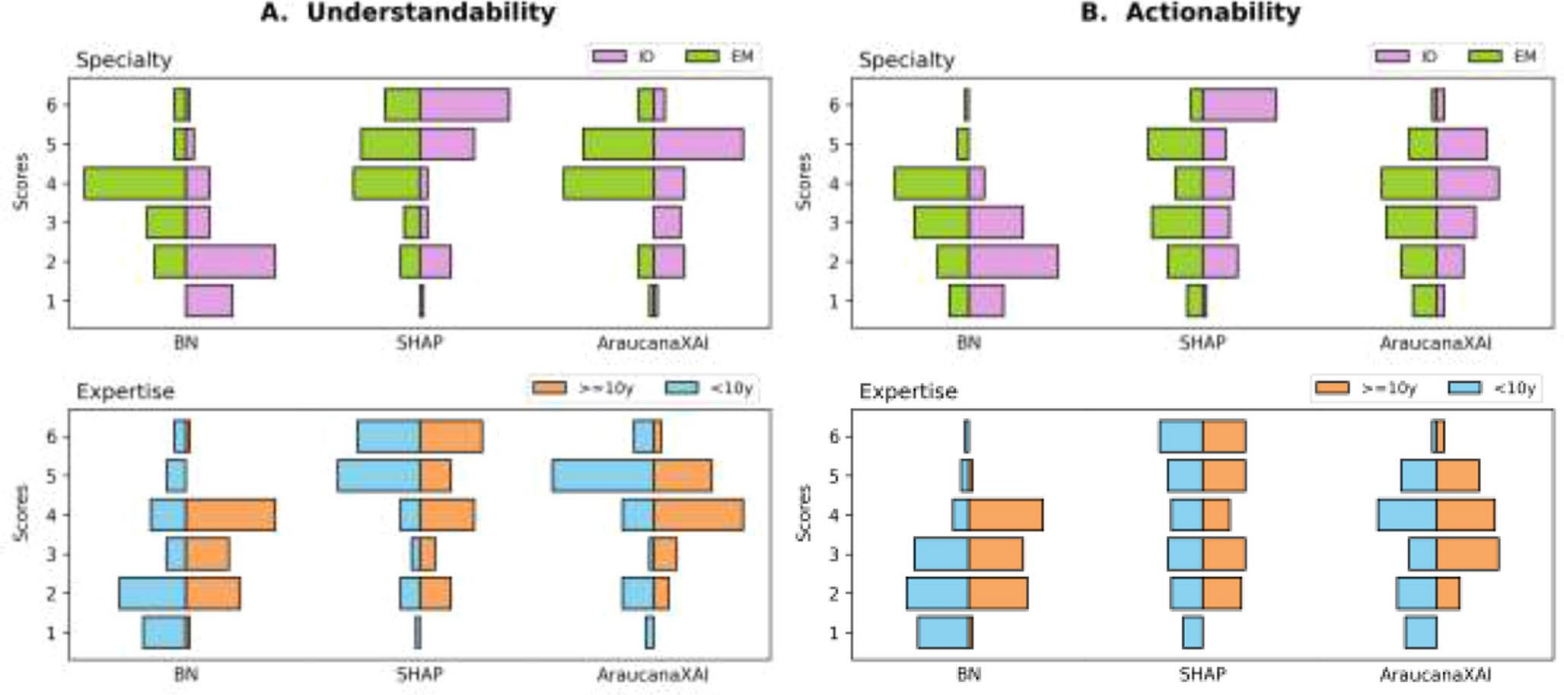
Ratings collected through the survey questionnaire. Count of (**A**) understandability and (**B**) actionability ratings stratified by specialty—ID vs. EM, and expertise—less vs. more or equal than 10 years of experience

Figure 3 shows an overview of the Likert ratings of each XAI method, collected through the *Survey Questionnaire*, stratified by specialty—ID vs. EM, and expertise—less vs. more or equal than 10 years of experience.

For RQ1, *understandability* and *actionability* dimensions are positively correlated (overall ρ > 0.45, p-values < 0.001), except in the AraucanaXAI results for EM specialty (ρ: 0.17, p-value: 0.23) and for clinicians with less expertise (ρ: 0.27, p-value: 0.06).

We conduct intra-pair comparisons to assess the similarity of *understandability* and *actionability* rates using weighted Cohen’s kappa statistic (results are provided in Supplementary Materials, Table S. 1). No clinician pair reaches the threshold of 0.4 [36] for any method (i.e. SHAP, AraucanaXAI, and BN), which indicates a moderate level of agreement. The absence of consistently high agreement suggests that clinicians may inherently differ in how they perceive and evaluate explanations.

**Fig. 4:**
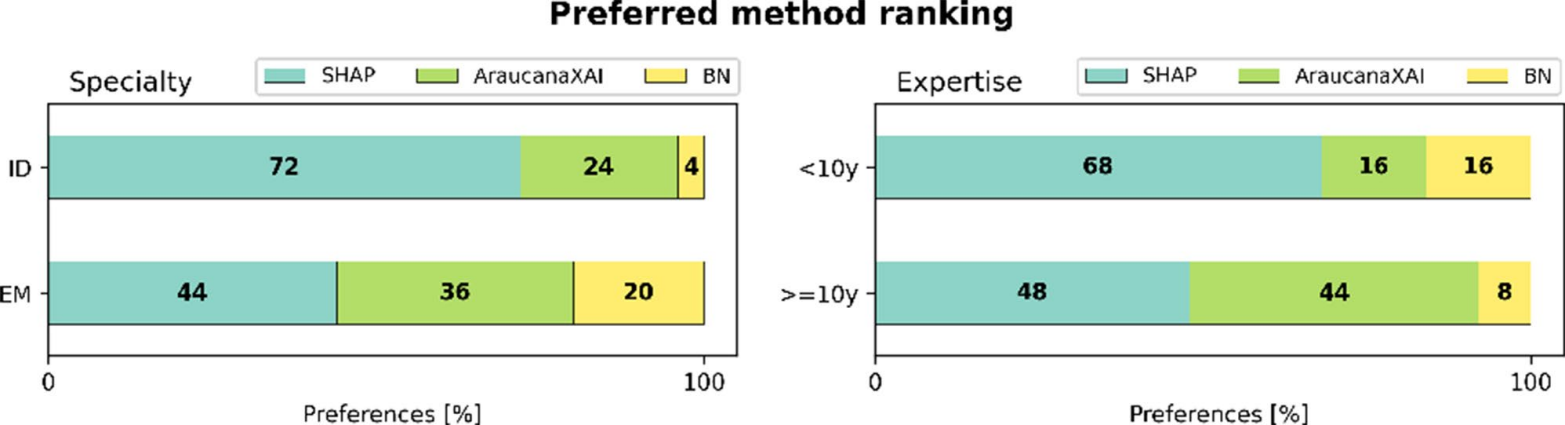
Clinicians’ preferred method ranking. Percentage of preferences associated to each method (SHAP, AraucanaXAI, and BN) stratified by specialty—ID vs. EM, and expertise—less vs. more or equal than 10 years of experience

For RQ2, there is a statistically significant difference in the BN results considering both the medical specialty (*understandability*: p-value < 0.001, and *actionability*: p-value < 0.05) and expertise (*understandability*: p-value < 0.05, and *actionability*: p-value < 0.001); instead for SHAP and AraucanaXAI, stratifications show no difference (*understandability*: p-value > 0.05, and *actionability*: p-value > 0.05). Overall, SHAP is the clinicians’ preferred method, as shown in Fig. 4. However, while 72% of ID clinicians prefer SHAP, only 44% of EM clinicians do, with 36% favoring AraucanaXAI. Also, more experienced clinicians show similar preference percentages (48% prefer SHAP and 44% AraucanaXAI).

**Fig. 5:**
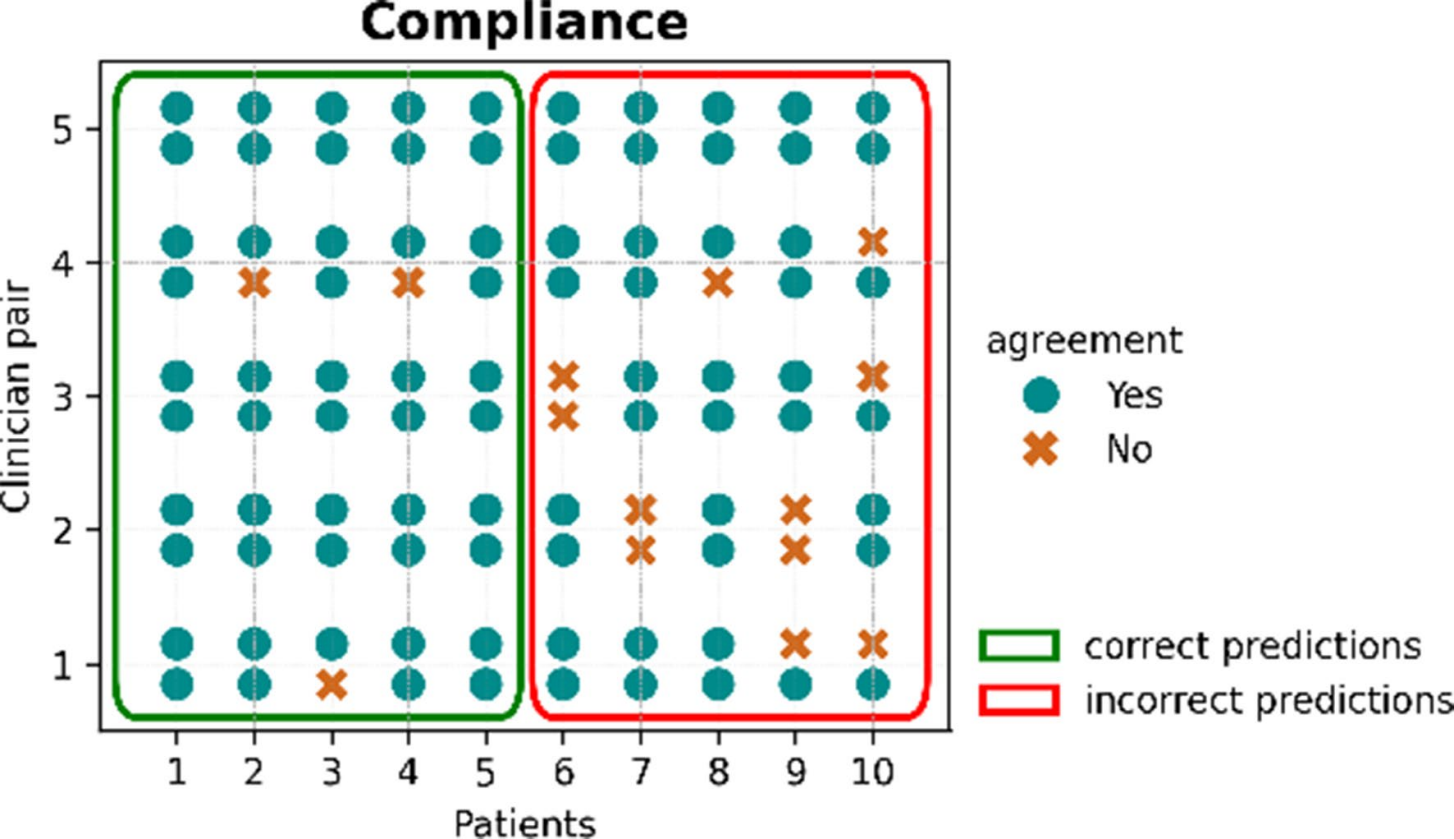
Overview of clinicians’ agreement with the predictions. Starting with patient 6, the cases move from TP and TN (correct predictions highlighted in the green box) to FP and FN (incorrect predictions highlighted in the red box)

In general, clinicians’ agreement with the AI prediction or *compliance* (Fig. 5) is high, ranging from 70 to 100% (86% on average), even though 50% of the presented cases have been purposefully chosen among the misclassified. This highlights a potential over-reliance effect that XAI may promote [8, 37]. When comparing compliance rates to years of experience, there is a general trend showing that compliance increases with expertise (Figure S. 1, Supplementary Materials). This pattern is consistent when stratifying by gender and specialty (as shown in Figure S. 1, Supplementary Materials); in this latter case, a strong link between greater experience and higher compliance is observed for ID, while the trend is much weaker for EM. Moreover, there is an inverse relationship between years of experience and the time taken to complete the survey, while a slight positive relationship exists between compliance and survey completion time (as shown in Figure S. 2, Supplementary Materials). In the end, considering clinicians attitudes towards AI (Figure S. 3, Supplementary Materials), less compliance (from 70 to 80%) is seen for those clinicians (3 out of 10) self-declaring to be familiar with AI, while the positive trend between higher compliance and more years of experience is associated with unfamiliarity with AI. On the other hand, the AI skeptic clinicians (2 out of 10, self-declared in the *Profiling Questionnaire*) have the same unexpectedly high compliance rate (90%).

### Think-aloud results

**Fig. 6:**
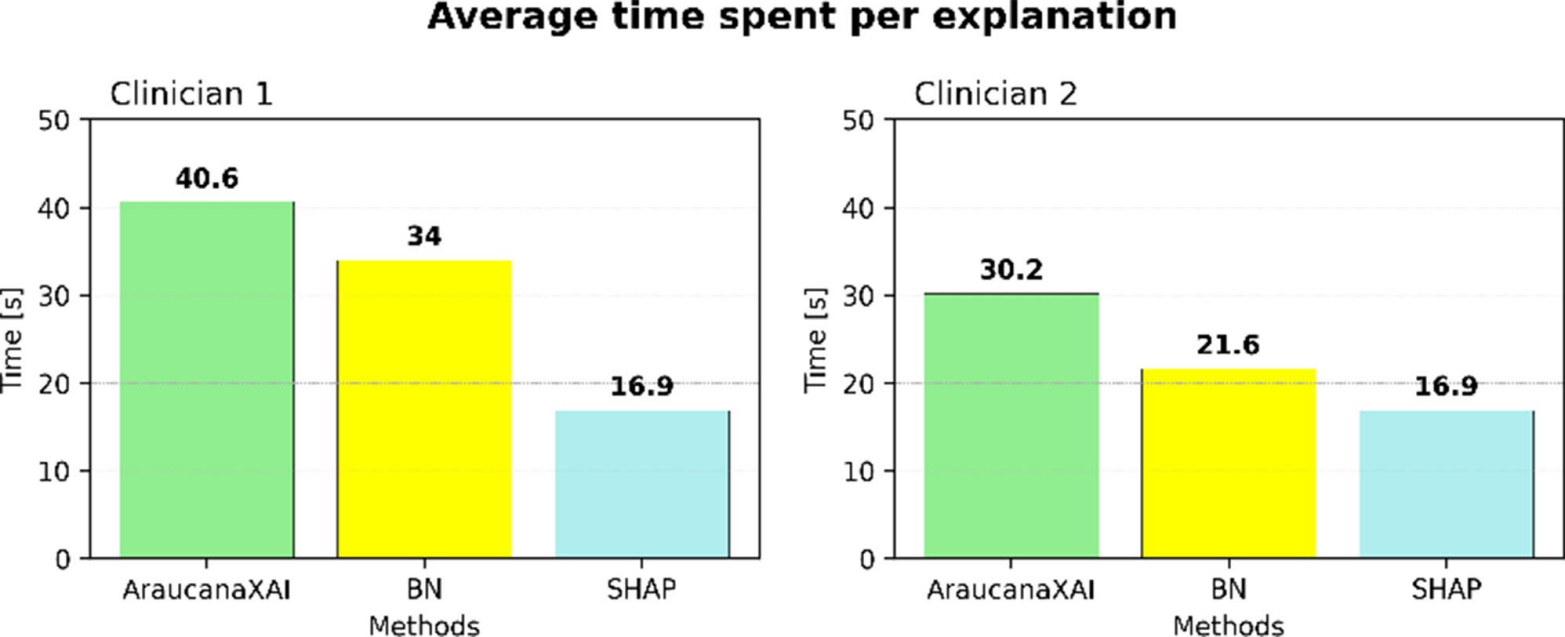
Average time spent per explanation during think-aloud interviews. The pattern of time spent on average on the methods is the same for both clinicians: more time is used for AraucanaXAI, followed by BN, and SHAP

The following analysis concerns the results obtained from the two think-aloud interviews. The interviewees are indicated as clinician 1 and clinician 2 and are respectively from EM (male, unfamiliar but with a positive attitude with AI) and ID (female, unfamiliar and skeptical towards AI), both with at least 10 years of experience. Figure 6 provides a general overview of the average time each interviewee spends observing and evaluating each explanation. A similar pattern is observed for both clinicians: the longest average time is spent on AraucanaXAI (over 30 s), followed by BN (between 20 and 30 s), while SHAP has the least average time, with both spending 17 s on average. Excluding SHAP, clinician 1 spends on average more time on the explanations than clinician 2.

**Fig. 7:**
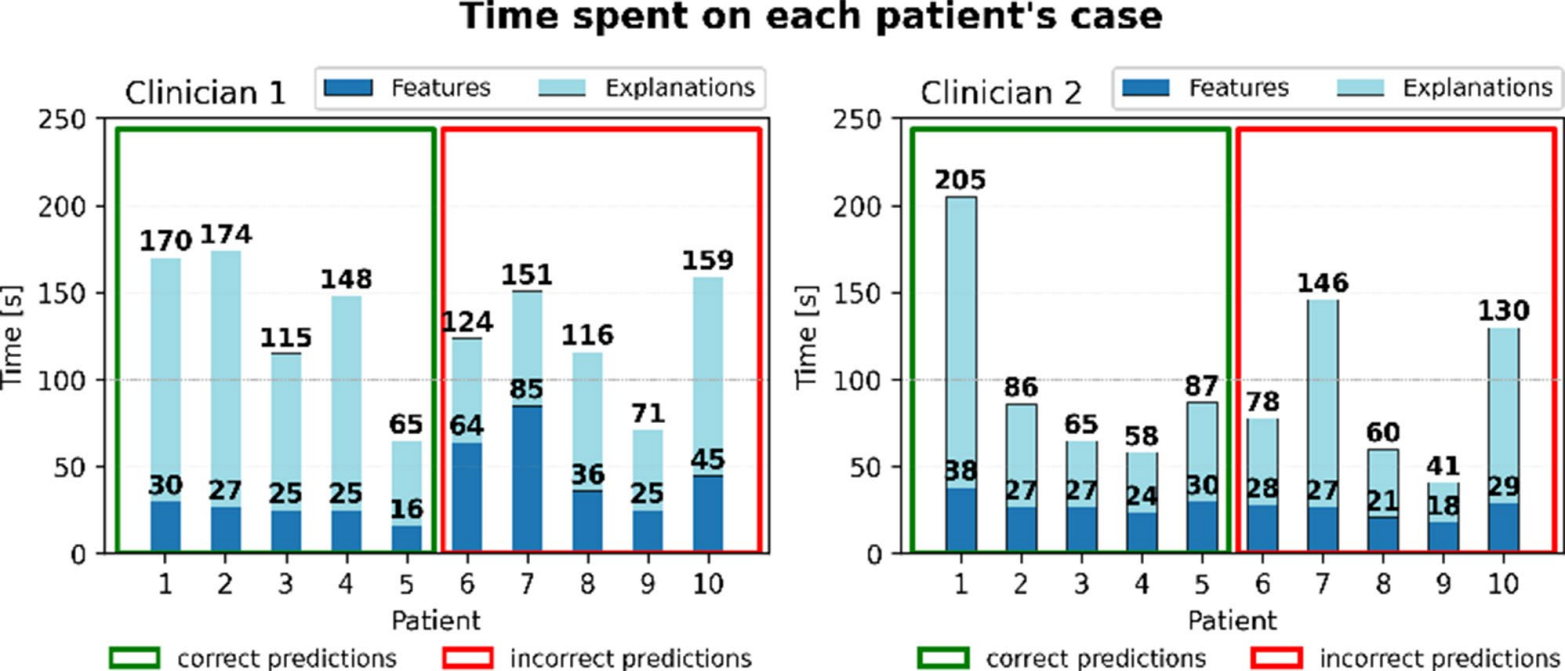
Time spent on each patient’s case during think-aloud interviews. The time is divided between looking at the patient’s features (in dark blue) and looking at all the explanations (in light blue), i.e. the sum of time spent on SHAP, AraucanaXAI, and BN, for each case. Starting with patient 6, the cases move from TP and TN (correct predictions highlighted in the green box) to FP and FN (incorrect predictions highlighted in the red box)

Additionally, Fig. 7 provides the time allocated by the two interviewees initially looking at each patient’s features and then looking at all the explanations. Clinician 1 spends more time initially looking at the incorrectly predicted cases (from patients 6 to 10), while a qualitative uniform pattern can be observed for clinician 2. In contrast, when considering the total time spent examining the explanations for each patient’s case, a more varied distribution is observed, with no clear informative pattern emerging. However, there is a notable peak in the time spent by clinician 2 to observe the first case, likely due to the need to interpret the explanation visualizations. This differs from clinician 1, who spends a more consistent amount of time examining each patient’s explanations.

Segmenting the interviews by explanation, the intra-clinician comparison shows a statistically significant difference only for clinician 1 for SHAP compared to BN (p-value < 0.05) and AraucanaXAI (p-value < 0.05).

## Discussion

In the context of the ALFABETO project, which employs ML classifiers to predict whether COVID-19 patients need hospitalization or home care, we conducted a user-centered study to collect clinicians’ perceptions of XAI and their preferences. We involved 10 experienced clinicians, stratified by specialty (ID vs. EM) and expertise (less vs. more or equal than 10 years of experience), collecting their cognitive evaluations of SHAP, AraucanaXAI and BN.

Like other previous similar studies [16, 18, 20, 38], we acknowledge the limited sample size (10 clinicians) as one of the main limitations of this work, and we recognize that the findings cannot be broadly generalized across all specialties or clinical settings. Furthermore, only two clinicians voluntarily participated in the think-aloud sessions, from which we derived the in-depth qualitative findings. Nevertheless, the key insights remain valid within the specific context described and provide a valuable foundation for future, larger-scale investigations. While this study focused on SHAP, AraucanaXAI, and the Bayesian Network, we are aware that other widely used XAI techniques—such as causal models, saliency maps (e.g., Grad-CAM), Layer-wise Relevance Propagation, or LIME—were not included in the evaluation. Our selection was guided by the tools available and the scope of the project, and although this introduces a degree of methodological bias (i.e. comparing *post-hoc* and inherently interpretable models), we believe the chosen techniques still offer meaningful insights into clinicians’ perceptions. Future research could expand on these findings by including a wider variety of explainability methods, further broadening the analysis and its applicability.

Here, our main goal is to assess clinicians’ attitudes towards various kind of XAI approaches, rather than validating ML classifiers previously developed as part of the ALFABETO project. As a matter of fact, we used ALFABETO as a case study to investigate XAI *understandability* and *actionability* based on real data from the same hospital of the involved clinicians.

The analysis of the *understandability* and *actionability* scores comparing SHAP, AraucanaXAI, and BN (Fig. 3), reveals several key implications for the use of different XAI methods in medical decision-making. Firstly, SHAP consistently scores highest in both cognitive dimensions, suggesting that its straightforward and simple visualization [39] makes it more understandable and actionable for clinicians. However, this ease of use may lead to over-reliance, particularly in complex, high-stakes decisions [37, 40]. In contrast, AraucanaXAI results show how it demands more effort leading to lower *actionability* scores than SHAP, but it encourages critical reasoning and may help mitigate over-reliance [37]. Nevertheless, the lowest *understandability* and *actionability* scores may indicate a negative bias towards the BN suggesting either potential limitations in its ability to provide clear and useful explanations or insufficient understanding due to inadequate instruction during the educational session [41]. Intra-clinician pair comparisons show very low agreement, falling below the threshold for moderate agreement. This highlights the inherent human subjectivity involved in interpreting and evaluating explanations, which appears to be influenced by individual and context-dependent goals [39, 42]. However, this comparison has limitations: the sample size of clinician pairs (5) may be insufficient for generalizing the results, and while weighted Cohen’s kappa statistic is useful, it may not fully capture the nuances of agreement and disagreement.

Clinicians preferences for explanation methods do not vary too much across specialties and expertise (Fig. 4). However, while the ID clinicians have a very strong preference for SHAP, EM clinicians show a more balanced preference between SHAP and AraucanaXAI. Indeed, the think-aloud interviewee from EM (clinician 1) remarkably appreciates how AraucanaXAI’s explanations mirror EM clinicians’ mental flowcharts (likely due to prior exposure to similar methods), which supports rapid decision-making—critical in emergency settings. On the other hand, ID clinicians face longer decision-making processes involving more factors, e.g. radiological images and direct patient interaction. The lack of face-to-face patient contact is identified as a limitation by the ID interviewee (clinician 2). These findings highlight the importance of clinicians’ prior experience and the complexity of selecting the most suitable XAI method [18, 39], which should be considered in future research.

A high level of compliance (86% on average [14, 35]), indicates that clinicians are relying too much on the predictions and the XAI explanations even when the AI prediction is wrong, potentially leading to overconfidence. This overconfidence occurs when the user places excessive trust in the system, resulting in agreement with incorrect system recommendations and therefore in lacking of critical evaluation of each clinical scenario [40]. This effect is usually classified as *automation bias* [43], i.e. “the tendency to over-rely on automation”. *Automation bias* can lead to significant errors, especially in high-stakes environments like healthcare. An important factor in the analysis of compliance is clinician expertise. Although the study sample is small (10 clinicians), there is a clear positive relationship between years of experience and compliance (Figure S. 1, Supplementary Materials). Task difficulty is another key factor. The inclination to over-rely on decision aids is influenced by the complexity of the task: as tasks become more challenging, there is an increased tendency to rely on decision aids (i.e. external sources) [44]. The think-aloud interviews suggest that incorrectly predicted cases are much more difficult to classify compared to the correct ones in this study, and, therefore, are likely to be labelled as borderline cases. However, the clinicians are not asked about the level of difficulty and stakes they have in mind while performing the tasks. Their subjective perception of task difficulty (i.e. complex vs. simpler case) and stakes could shed more light on the high compliance rates [16]. For instance, agreement with predictions in ambiguous cases, where classification is uncertain, carries less weight than agreement in cases of clear misclassification. A more comprehensive evaluation could be achieved by considering the perceived difficulty of each case and clinicians’ confidence in their decisions [16]. To better understand compliance, future studies should improve the design by collecting clinicians’ decisions both before and after they view each XAI method [19]. This would also enable alternative agreement assessment approaches, such as clustering clinicians based on rating strategies [45]. The current study cannot fully assess the differences between the three methods, as clinicians are only asked to indicate agreement with predictions after examining all three explanations for each patient.

The differences in day-to-day decision-making processes between the two departments should be taken into account during the interpretation of the results and while developing XAI for decision support systems (DSSs). The analysis of time spent on each explanation, alongside initial patient observation, offers valuable insights into the decision-making process. The average time spent on each explanation reveals distinct patterns in the clinicians’ approaches. Both clinicians allocate the least amount of time on SHAP explanations, the most on AraucanaXAI, with the BN falling in between (Fig. 6). The BN is often skipped due to its lack of *understandability* and, consequently, its lower *actionability*. More complex methods, like the BN, require more time to extract meaningful insights, whereas AraucanaXAI, though understandable, still requires more time than SHAP. Additionally, the initial time spent looking at the patients’ features varies significantly between clinicians (Fig. 7). Clinician 1 spends more time initially observing patients’ features, especially from patient 6 onward, while clinician 2 maintains a consistent uniform distribution of initial observation times. This difference may reflect variations in their diagnostic strategies. The statistically significant results for clinician 1’s SHAP explanations compared to BN and AraucanaXAI highlight SHAP’s ease of use. Both clinicians generally spend enough time on incorrectly predicted cases (Fig. 7), but this extra time does not lead to prediction disagreement, with both showing high compliance (90%).

It is important to note that the time measurements are taken during the think-aloud session, while clinicians are encouraged to deliberate and express their thoughts, which sometimes causes overlap in explanations and makes setting precise time boundaries difficult. Neutral or general comments are also interspersed throughout the session. Time measurements of a separate group of clinicians, conducted without the think-aloud interview, could provide a more objective assessment of time allocation and cognitive load.

## Conclusions

The combination of survey data and think-aloud protocol provides a robust understanding of clinicians’ interactions with XAI tools, highlighting both their strengths and areas for improvement. While the survey captures clinicians’ quantitative evaluation of the methods (i.e. SHAP, AraucanaXAI, and BN), the think-aloud sessions reveal their reasoning and measure cognitive load by tracking time.

In this study, clinicians have a generally positive attitude towards AI, resulting in trust in the system and its explanations. However, a key challenge remains in addressing human-AI disagreements, as indicated by the high compliance rates (86% on average). These latter suggest clinicians may over-rely on AI predictions when coupled with XAI, further increasing the risk of *automation bias*, particularly in complex cases where critical evaluation is crucial.

Answering RQ1, comparisons of the XAI explanations reveal a strong correlation between the two cognitive dimensions investigated—*understandability* and *actionability*. The variability in intra-clinician pair comparisons suggests that agreement on *understandability* and *actionability* scores may be influenced by unmeasured factors.

Answering RQ2, there are noticeable differences between the methods (i.e. SHAP, AraucanaXAI, and BN) depending on expertise, specialty, and the setting where the clinician makes decisions. SHAP is generally rated as the most understandable and actionable, while BN scores significantly lower across all measures. The study suggests that improving BN’s *understandability* would require better design and additional educational training, as it demands more cognitive effort and time compared to more intuitive and self-explanatory methods like SHAP and AraucanaXAI.

These results underscore the importance of selecting the appropriate XAI method based on the specific requirements of the clinical context and medical user, such as specialty, expertise, decision stakes, time constraints, and workload, to name a few. The statistically significant higher scores for SHAP and AraucanaXAI indicate they are strong candidates for enhancing reliance and usability in XAI systems, ultimately improving clinicians’ efficiency. Ongoing research and evaluation are needed to refine these methods and ensure their continued effectiveness as standards of care evolve, particularly with larger sample sizes.

## Electronic supplementary material

Below is the link to the electronic supplementary material.

Supplementary Material 1

## Data Availability

The read-only version of the web-based questionnaires used in the experiment described in this paper are available at the following links: Profiling Questionnaire– https://ee.kobotoolbox.org/preview/xcdRzrfQ, Survey Questionnaire– https://ee.kobotoolbox.org/preview/aSyf4ED2. The dataset generated by the survey, that support the findings of this study, is available from the corresponding author upon reasonable request.

## Abbreviations

XAI: eXplainable Artificial Intelligence
DSS: Decision Support System
AI: Artificial Intelligence
ML: Machine Learning
ID: Infectious Disease
EM: Emergency Medicine
TP: True Positive
FP: False Positive
TN: True Negative
FN: False Negative
BN: Bayesian Network
ESS: Explanation Satisfaction Scale
