# Supplementary material for: Which explanations do clinicians prefer? A comparative evaluation of XAI understandability and actionability in predicting the need for hospitalization
Source: BMC Med Inform Decis Mak. 2025 Jul 16;25:269. doi: 10.1186/s12911-025-03045-0 (PMC12265231; doi:10.1186/s12911-025-03045-0)
Supplement: Supplementary file 1 — Supplementary Material 1 [file 12911_2025_3045_MOESM1_ESM.docx]

Supplementary Materials

Profiling Questionnaire

The *Profiling Questionnaire* administered to each survey participant is shown below. It collects some profiling information, specifically years of experience as medical specialist, specialty, gender, and attitudes toward AI (yes/no statements from 1 to 5).

Survey Questionnaire

A read-only version of a *survey instance*, i.e. patient case, of the *Survey Questionnaire,* is shown below.

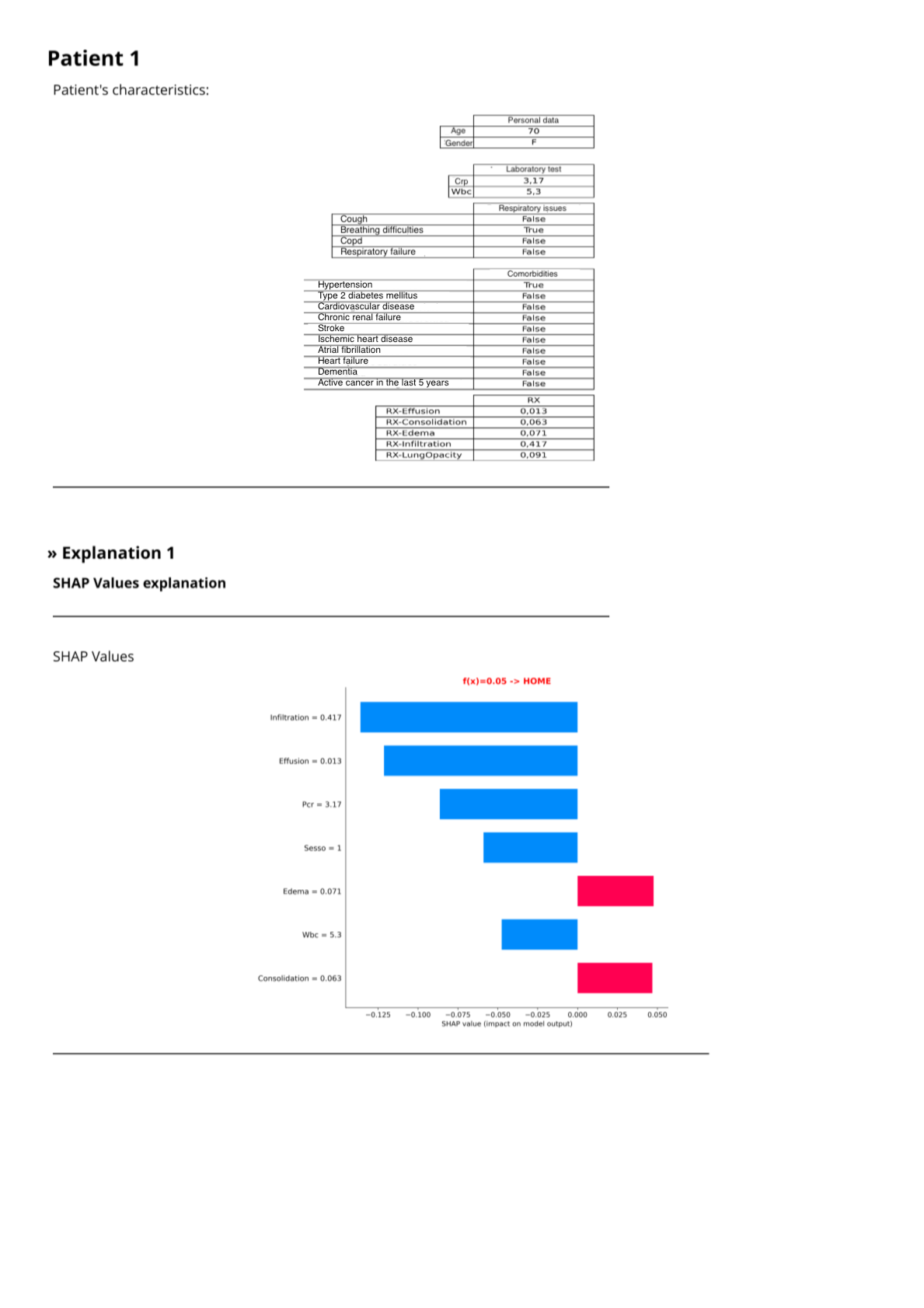


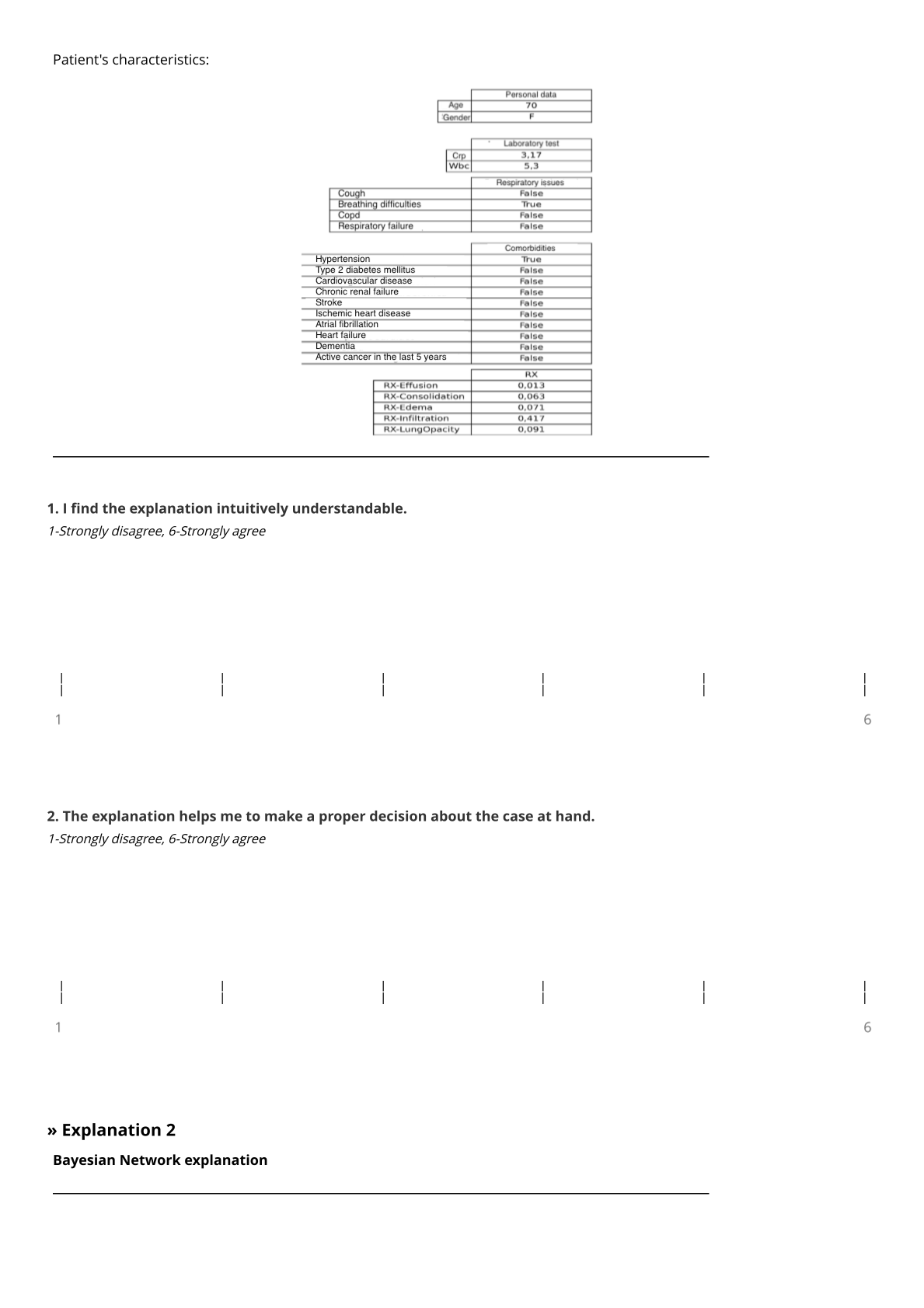

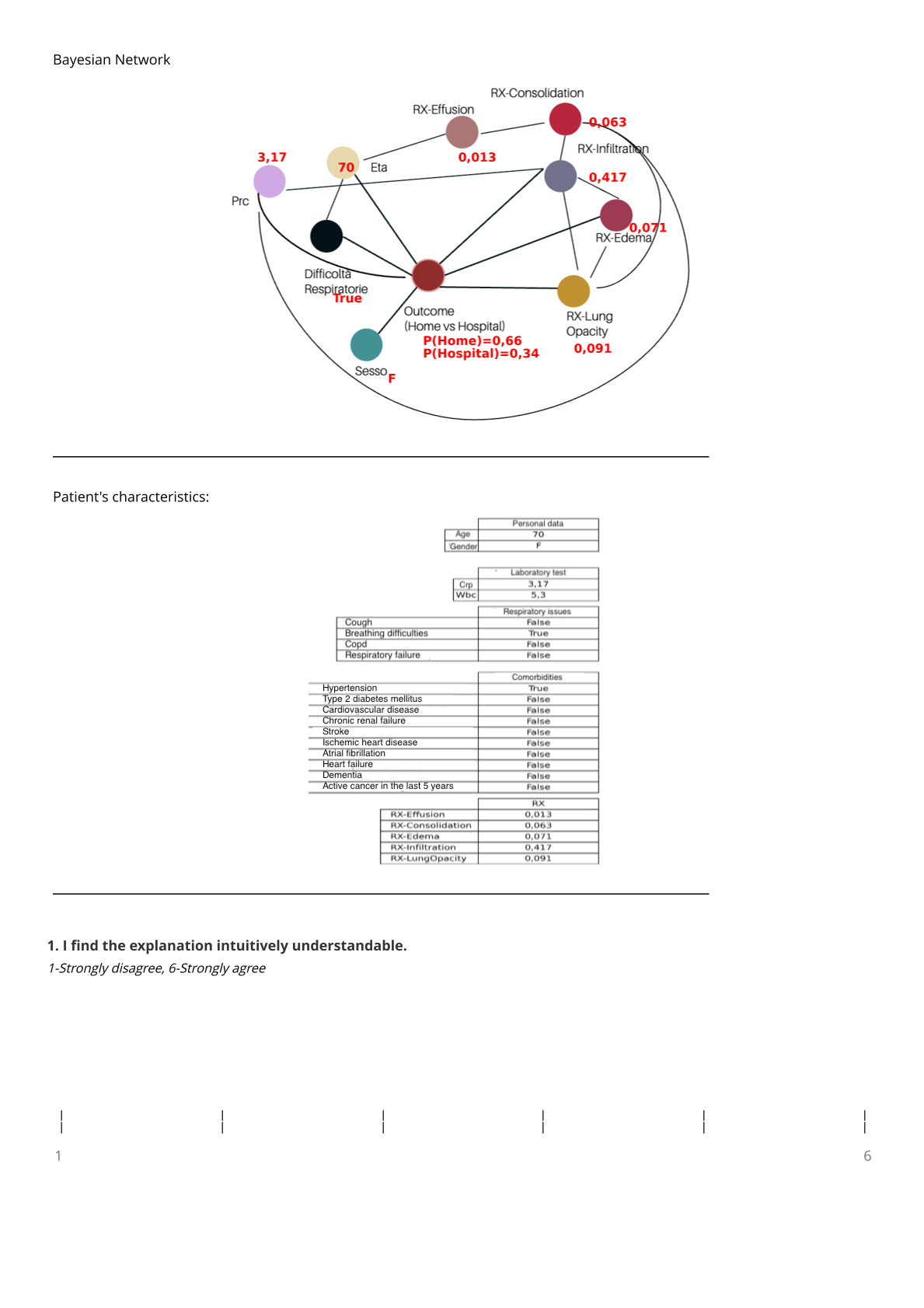

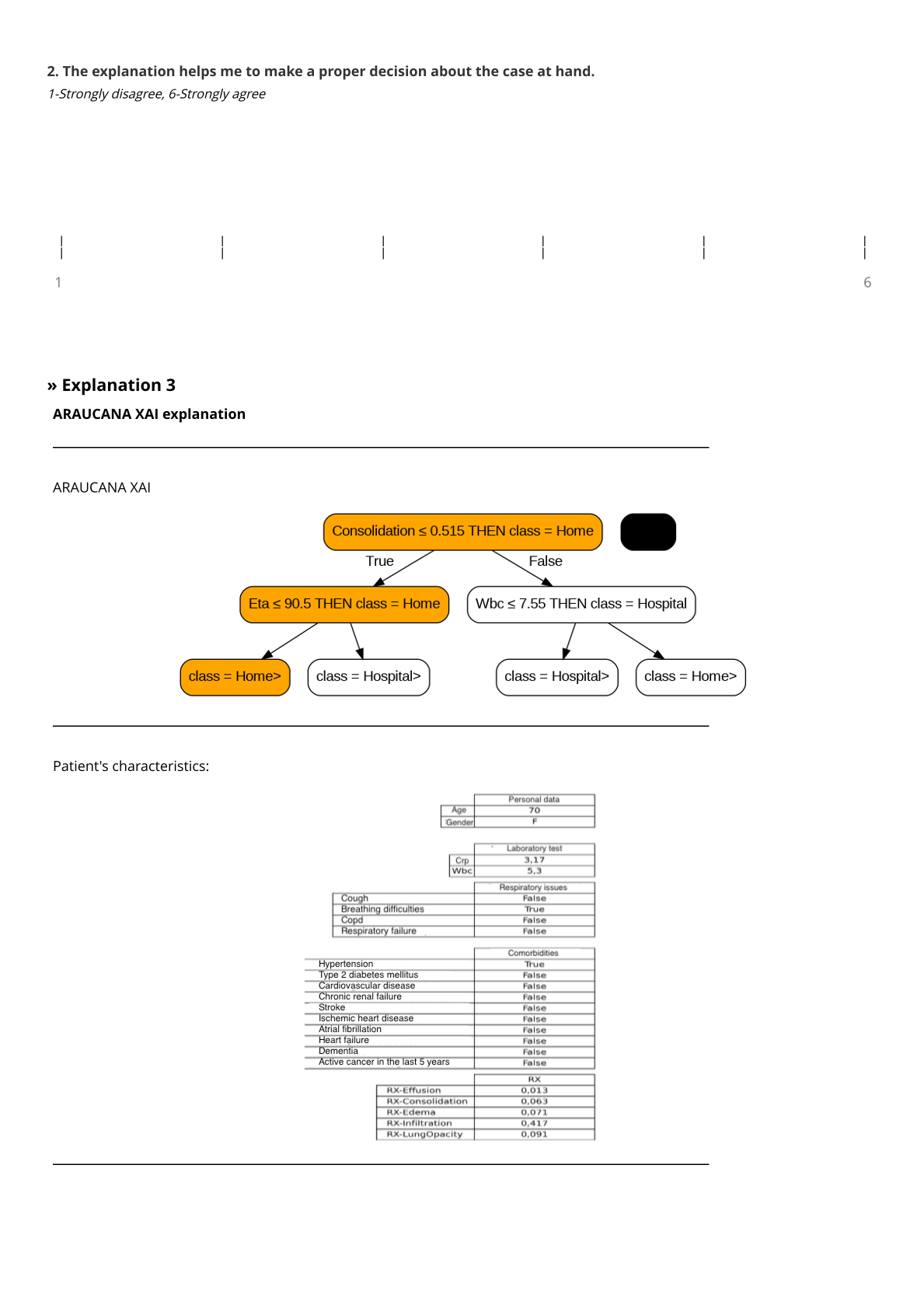

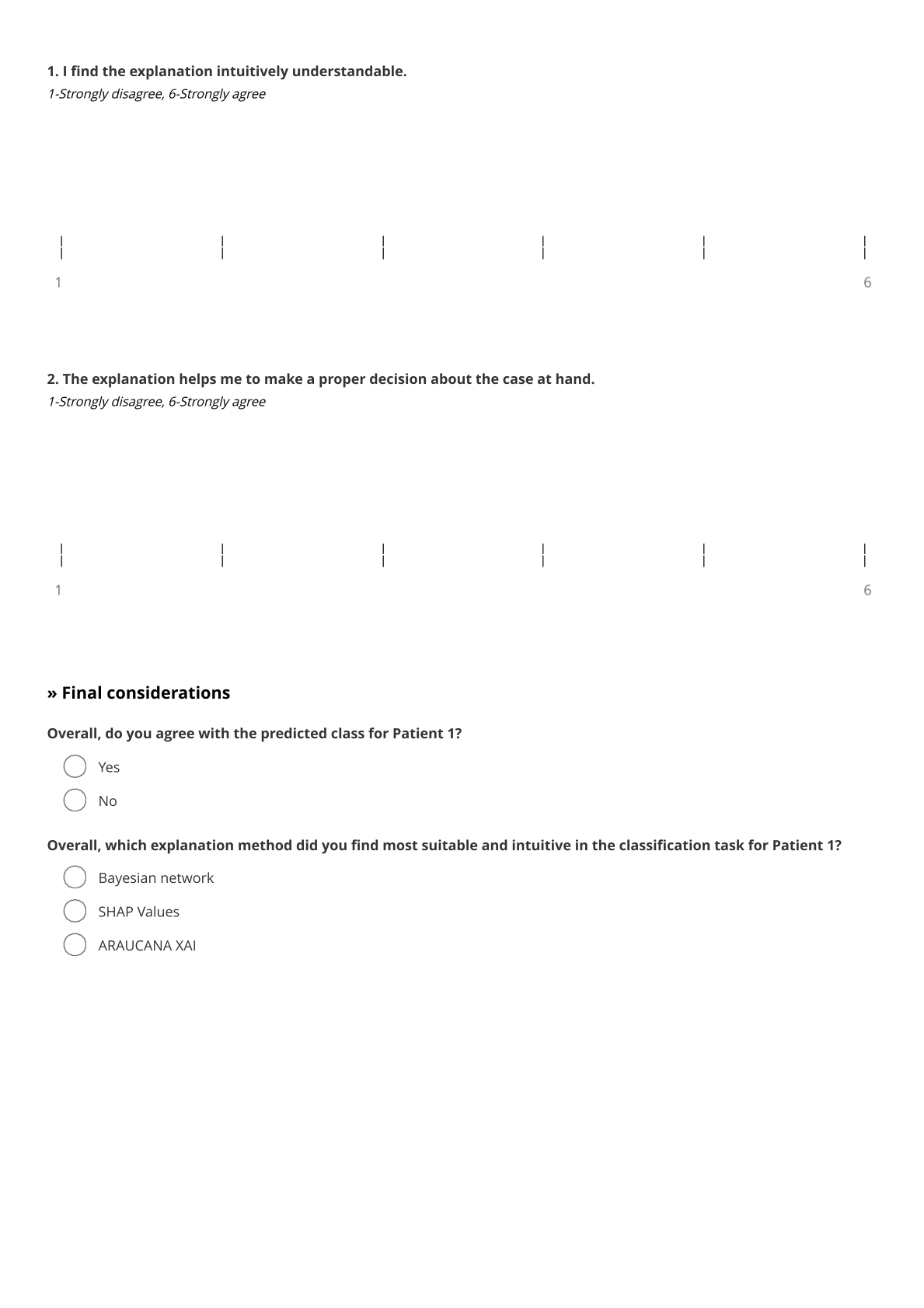


Additional survey analyses

In the study, the average compliance obtained is relatively high (equal to 86% on average), especially taking into consideration that half of the presented cases are incorrectly predicted (FP and FN). When comparing compliance rates and the years of experience, we can observe a positive trend showing that compliance increases with expertise (Figure S. 1, left). The same pattern can be seen also stratifying by gender (Figure S. 1, center) and specialty (Figure S. 1, right). Stratifying by specialty, a strong relationship between greater experience and higher compliance is observed for ID, while the trend is much weaker for EM.


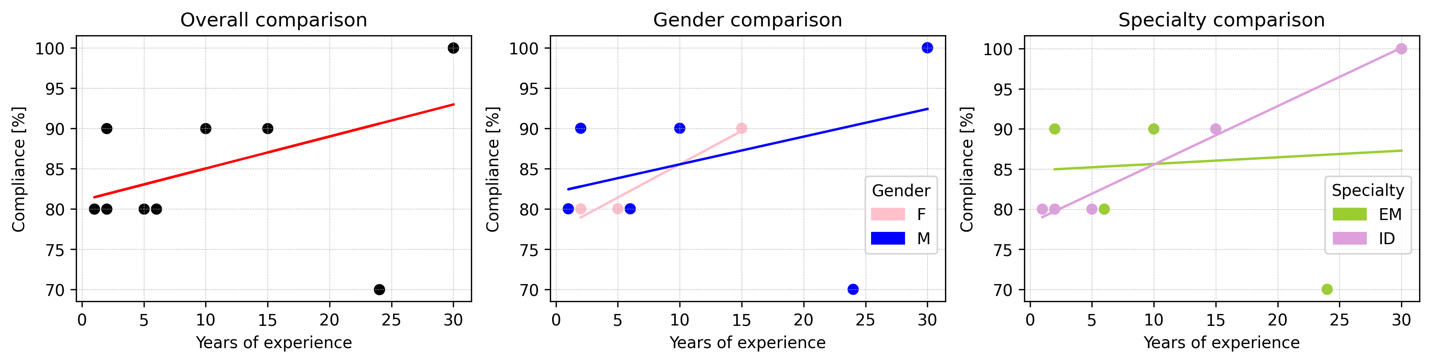


Figure S. 1 Stratified-comparisons of compliance rates with clinicians’ years of experience. The comparisons are shown with no (left), gender (center) and specialty (right) stratifications respectively.

During the survey, we also record the time each clinician spends completing the *Survey Questionnaire*. The time is measured from when the clinician first opens the questionnaire link until he/she submits his/her responses (after having seen all 10 patients’ cases). Overall, survey completion time ranges from 6 to 85 minutes, with an average of 29 minutes. The time just described can be compared either with the years of experience of each clinician or with the compliance rates. In the first case (Figure S. 2, left), we can see that there is a negative relationship between time and years of experience, whereas in the second case (Figure S. 2, right) a slightly positive relationship exists between compliance and time.


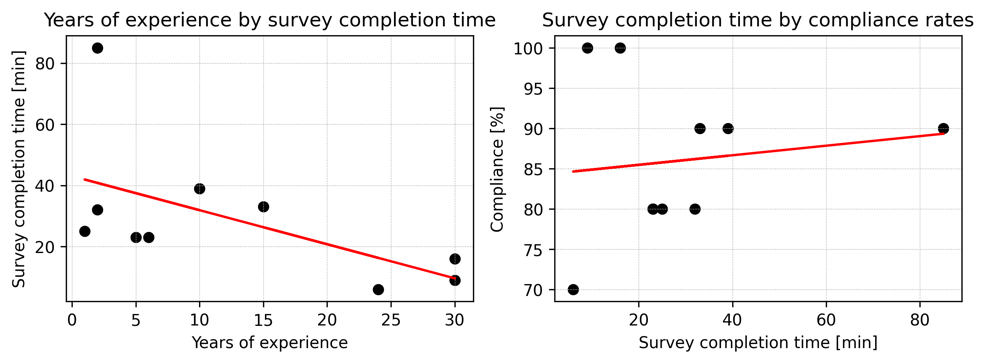


Figure S. 2 Comparison of survey completion time with years of experience (left) and compliance (right).

Finally, we can consider compliance and years of experience to clinicians’ attitudes towards AI. Indeed, as shown in Figure S. 3 (left), clinicians familiar with AI show lower compliance rates (from 70% to 80%), while unfamiliar ones are more compliant (from 80% to 100%). On the other hand (right), being skeptical was not associated with different compliance rates (both skeptic clinicians have 90% of compliance). The two dimensions just examined, i.e. familiarity and skepticism, do not reveal any specific trends related to years of experience.


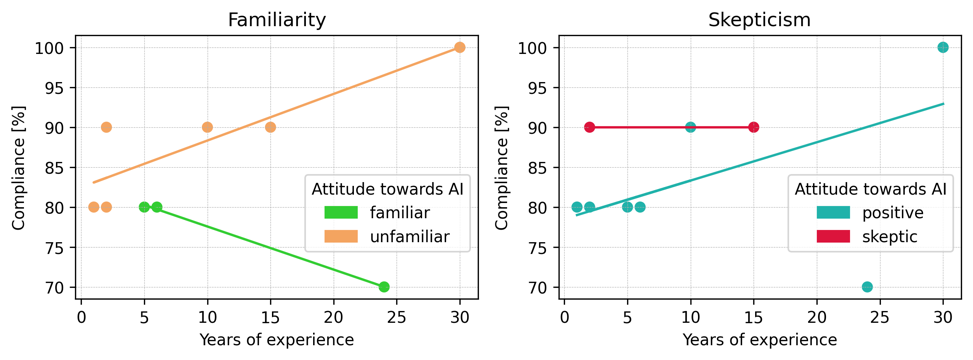


Figure S. 3 Compliance and years of experience in the light of clinicians’ attitudes towards AI. Attitudes towards AI have been distinguished as familiarity (left) and skepticism (right).

*Intra-pair comparison*

In order to systematically test the similarity of *understandability* and *actionability* rates, weighted Cohen’s kappa is calculated. The scores obtained are compared with the reference from the literature (Perfect agreement: k=1; Excellent agreement: k>0.75; Moderate to good agreement: 0.4<k<=0.75; Poor agreement: k<=0.4; Agreement worse than chance: k<0). None of the scores reached the 0.4 threshold which is considered a moderately good agreement. The highest obtained score is 0.35 for the *actionability* of SHAP for pair 3, as shown in Table S. 1.

Table S. 1 Cohen’s kappa inter-pair agreement for actionability and understandability dimensions.

| **Cohen’s kappa inter-pair agreement**  **actionability** | | | | **Cohen’s kappa inter-pair agreement**  **understandability** | | | |
| --- | --- | --- | --- | --- | --- | --- | --- |
| **Pair** | **SHAP** | **AraucanaXAI** | **BN** | **Pair** | **SHAP** | **AraucanaXAI** | **BN** |
| **1** | -0.01 | 0.04 | -0.06 | **1** | -0.07 | 0.07 | 0.07 |
| **2** | -0.2 | -0.11 | -0.13 | **2** | -0.39 | -0.28 | 0 |
| **3** | 0.35 | -0.3 | -0.04 | **3** | -0.06 | 0 | 0.02 |
| **4** | 0.01 | -0.04 | 0.33 | **4** | 0 | 0 | 0 |
| **5** | -0.1 | 0.01 | 0 | **5** | -0.06 | -0.02 | 0 |
